# Activated Carbon Modifications for Heterogeneous Fenton-Like Catalysis

**Published:** 2022-06-10

**Authors:** P Compton, NR Dehkordi, P Larese Casanova, AN Alshawabkeh

**Affiliations:** Department of Civil and Environmental Engineering, Northeastern University, Boston, MA, USA

**Keywords:** Heterogeneous catalysts_1_, oxidation processes_2_, activated carbon_3_, Fenton_4_, manganese oxides

## Abstract

The effective and efficient degradation of persistent, recalcitrant pollutants by advanced oxidation processes is vital to both reduce hazardous waste and remediate polluted waters. One such advanced oxidation process is the use of Fenton chemistry, which can be optimized using heterogeneous catalysts. However, to make this AOP viable over conventional treatment methods, the technology needs to be optimized from both a technical and economic standpoint. From a heterogeneous catalyst optimization perspective, varying the surface chemistry of activated carbon and impregnating or doping with Fenton-like catalytic nanomaterials removes precipitation complications associated with traditional iron species in Fenton chemistry while generating effective amounts of highly oxidative hydroxyl radicals. Utilizing various techniques to synthesize heterogeneous catalysts with activated carbon as a backbone, in the presence of H_2_O_2_ the formation of hydroxyl radicals and removal of benzoic acid is tested. Comparing various additives, raw activated carbon impregnated with 5% MnO_2_ in the presence of H_2_O_2_ realized a high concentration of hydroxyl radical formation while maintaining low cost and relative ease of synthesis. This AC-Mn5 catalyst performed effectively in varying concentrations of H_2_O_2_, utilizing various synthesis techniques, after simulated aging of the catalyst structure, and over a wide pH range with the highest radical formation at acidic pH values. Utilizing this catalytic material as a substitute for iron species associated with traditional Fenton technology, the goal of designing a full set of oxidation functions towards persistent, recalcitrant pollutant removal while maintaining cost-effectiveness and scalability is proposed. It is anticipated these catalytic materials are effective to eliminate analogous contaminants and mixtures.

## Introduction

The widespread contamination of water bodies by various pollutants requires novel techniques in not only determining and quantifying their concentration, but also in removal of these contaminants from aqueous matrices to protect human health and the environment. Contaminant classes range from heavy metals, anionic toxic species, dyes, pharmaceutical active compounds, pesticides, and others that come from a wide array of sources. Hexavalent chromium, which can be released from sources that conduct metal finishing, wood preserving, and petroleum refining, as well as mercury released from ferrous metals production and incineration processes can exist as highly toxic forms like Cr(VI) and Hg(II) and require novel techniques – such as electrochemical methods – to determine their presence in aqueous solutions [1,2]. Nitrate, while an essential nutrient for microorganism protein synthesis, can be considered toxic and cause eutrophication at higher concentrations in areas of agricultural runoff; newer electrochemical methods exist for nitrate determination with additional removal capabilities of these kinds of inorganics [3]. In addition, organics such as dyes and pharmaceutical compounds that are highly toxic and not easily biodegraded require innovative techniques – such as photodegradation processes - for their degradation and removal from water and wastewater [4,5]. These novel techniques of contaminant determination and removal, with a focus on ease of synthesis and operation, sustainability of materials, and scalability, are extremely important to address the growing ubiquity of newer classes of resilient contaminants.

The emergence of new organic, persistent pollutants in industrial and municipal wastewater is a problem for currently applied physical and chemical water treatment processes. These recalcitrant contaminants are extremely resistant to these generally applied processes [6] and require innovative methods, such as advanced oxidation processes (AOPs) to degrade and mineralize these highly stable compounds in wastewater. While the use of biodegradation on these recalcitrant pollutants is still feasible, tailoring and optimizing biological treatment for each specific organic compound is not viable for non-selective targeting [7]. As an option, AOPs are efficient and economical technologies to meet this emerging class of pollutants and provide for non-selectivity in treatment.

While there are many advanced processes to degrade and remove recalcitrant contaminants [8], one incredibly powerful AOP is the Fenton process that uses fundamental Fenton chemistry to produce hydroxyl radicals, which are highly potent oxidizing agents. For over a century, scientists and engineers have known the effects of mixing Fe(II) and H_2_O_2_ to produce a strong oxidizing environment [9]. Fundamentally, Fenton chemistry involves the oxidation of Fe(II) to Fe(III) by H_2_O_2_, which also produces a hydroxide ion and hydroxyl radical. These hydroxyl radicals are extremely powerful oxidants which contain a highly unstable unpaired electron that can non-selectively oxidize surrounding compounds (i.e., recalcitrant pollutants). While this provides AOPs an advantage over biological treatment, there are still limitations with utilizing Fenton’s reaction for water treatment. These limitations include the precise pH regime in which Fe(II) and Fe(III) remain as free ions, the cost and logistics of using external H_2_O_2_, and the economic design of a usable water treatment reactor utilizing this technology [10]. Therefore, researchers and engineers have created optimization methods attempting to remove the constraints associated with Fenton chemistry for water treatment.

Three of the single optimization methods to the Fenton process to reduce constraints are heterogeneous Fenton, photo-Fenton, and electro-Fenton. Where the traditional Fenton process utilizes external addition of iron catalysts, heterogeneous Fenton and Fenton-like techniques apply solid catalysts containing active catalytic sites where Fenton-like reactions can occur [10]. The photo-Fenton optimization process employs varying wavelengths of light to aid in the reduction of Fe(III) to Fe(II) which can minimize iron sludge production and limit the amount of initial iron catalyst needed. Creating H_2_O_2_ by electrochemically reducing oxygen on a cathode as well as possibly reducing Fe(III) to Fe(II), the electro-Fenton process removes the need of external H_2_O_2_ and reduces iron sludge [11,12]. Indeed, each method has its own advantages and disadvantages regarding limited pH range efficacy, logistics of suitable materials, and economy of scale, but improvements to these processes has led to vast reductions of the constraints of Fenton technology.

While the advantages of heterogeneous Fenton focus on the pH regime problem of tradition Fenton chemistry, the drawbacks of this optimization are both economic and secondary contamination related. One such optimization utilizes Fe_2_O_3_ impregnated graphene oxide for use as a heterogeneous Fenton catalyst to widen the working pH range of the Fenton process as well as create a stable/reusable catalyst [13,14]. Other methods of implementing stable heterogeneous Fenton catalysts include Cu-Fe bimetallic catalysts impregnated over silica supports [15], CuFeZSM-5 zeolite catalysts [16], and iron species immobilized on clay plates [17] which also widen the working pH range and cause low iron sludge production.

These iron-containing heterogeneous catalysts have been implemented in water treatment systems to address many different types of organic contaminants. Furfural, a chemical feedstock and potential carcinogen, within a wastewater sample has been treated via photodegradation with iron oxide supported onto a clinoptilolite as a heterogeneous catalyst [18]. Suitable supports can significantly enhance the reactivity of the heterogeneous catalysts, as these clinoptilolite nanoparticles modified with iron oxides have also shown great efficacy in the photocatalytic degradation of the persistent antibiotic cotrimaxazole [19] and 2,4-dichloroaniline [20]. Indeed, varying iron oxides and/or other catalytic materials in combination with numerous suitable supports have highly unique catalytic properties which can be enhanced for use in degradation technologies. While low-cost ZnO nanoparticles have stable physicochemical properties but limited in photodegradation processes due to low-yield responses to visible light, in combination with α-Fe_2_O_3_ which can absorb a broad spectrum of light, enhanced photodegradation and mineralization of a dye can be achieved [21]. This synergistic effect with α-Fe_2_O_3_ has also shown efficacious with photocatalytically active p-type Cu_2_O for methylene blue degradation [21]. Most importantly, these iron oxide containing catalysts supported on backbones such as clinoptilolite or zeolite P have been effective in enhancing the degradation of real-world fish pond waste and recalcitrant chlorophenols, though as with all setups the efficacy is strongly dependent on pH, catalyst concentration, iron oxide loading, and the presence of parasitic anions [22,23]. These innovations are highly advantageous, but can accrue high synthesis costs [24], have negative synthetic conditions of catalyst and supports [25], and diminished promise for industrial use [26].

Therefore, it is essential that the research pathway to produce Fenton-like reactions utilizing heterogeneous catalysts focuses on materials and supports that are environmentally friendly and relatively inexpensive. Additionally, these catalysts must be designed to overcome traditional Fenton limitations such as the small pH range that limits large-scale application of the Fenton processes, while removing the need to recover precipitated catalytic materials following treatment and removing the ability of ion-complexing agents such as phosphate to deactivate the catalyst [27]. As a widely used adsorbent, granular activated carbon (GAC) can support a wide array of heterogeneous catalytic materials while also remaining relatively inexpensive [28]. As well, the surface chemistry of GAC can be modified using various techniques to introduce elements which increase both adsorption capacity and catalytic activity [29]. In the present study, modified GAC and GAC impregnated with various heterogeneous catalytic compounds were utilized and compared to determine the formation of hydroxyl radicals. Since GAC has robust adsorptive capabilities, the use of benzoic acid (BZA) as a model pollutant allows a quantitative determination of hydroxyl radical generation. There is a linear correlation between the formation of 4-hydroxybenzoic acid (4-HBA) and the concentration of hydroxyl radicals generated from the catalytic wet peroxide oxidation of BZA [30]. Various techniques were utilized to modify raw GAC, to include oxidizing activated carbon with concentrated nitric acid, doping this oxidized activated carbon (ACox) with various functional groups, and impregnating AC and ACox with certain heterogeneous catalysts, particularly synthesized iron oxychloride nanosheets and manganese oxide nanoparticles. The relative formation of hydroxyl radicals was determined to determine which AC modification resulted in the highest catalytic performance. In addition, special attention was paid to the ease, time, and cost of each AC modification and weighed alongside catalytic performance to determine the best candidate for further research. This follows the research pathway stated above and attempts to satisfy concerns with utilizing heterogeneous catalysts for Fenton-like oxidation.

### Experimental

#### Activated Carbon Modification

Raw −20+40 mesh activated carbon was purchased from Fisher Scientific. Two distinct methods were utilized to either dope or impregnate this AC or oxidized AC (ACox) with various catalysts. The doping method, detailed more extensively in [29], initially involves washing the raw AC with deionized (DI) water to remove adsorbed residuals and drying in an oven at 110 °C for 24 hrs. The first modification performed was a concentrated acid oxidation of this AC. This was accomplished by combining 5 grams of the washed AC to 20 mL of a concentrated nitric acid (~1.2 pH) and DI water solution with a 1:1 ratio. The mixture was stirred for several minutes, and then left suspended for 72 hours with periodic stirring. After settling, the solids were recovered with filtration and washed with DI until the filtrate pH was near neutral. These solids were then dried in an oven at 110°C for 48 hours, resulting in the oxidized AC (ACox). Next, AC and whichever chemical utilized to dope the activated carbon (i.e., 3 grams of urea for N-doping) was placed in a vessel and sonicated in DI water for 0.5 hrs. The modified carbon was then filtered, dried in an oven at 110 °C for 24 hrs, heat treated in a tube fur nace under a flow of Argon for 3 hrs at 900 °C at a heating rate of 10 °C/min, and finally cooled to room temperature. This method details the preparation for the doped catalysts utilized.

The second method to modify the AC involved impregnating either AC or ACox with catalytic materials. First, a catalytic material – iron oxychloride or manganese oxide nanopowder - was placed in 30 mL solvent (either DI water, acetone, or ethanol), and sonicated for 10 minutes. The mass ratios varied for each, with the names of each varying catalyst containing the mass ratio of catalytic materials to activated carbon. Either AC or ACox was then added, and the mixture was vortexed rapidly for 10 minutes. This mixture was then sealed and rotated for at least 48 hours, filtered, and then dried in an oven at 110 °C for 24 hours. This method details the preparation for the impregnated catalysts utilized.

#### Catalyst description

The three catalysts utilized to either dope or impregnate AC were nitrogen (by urea doping), manganese oxide (MnO_2_) and iron oxychloride (FeOCl). AC doped with urea has been shown to be more effective at benzoic acid (BZA) removal than parent activated carbon [29]. These nitrogen groups produce enhanced electron density on the carbon surface which increases both adsorption and catalytic activity. Heterogeneous FeOCl nanosheet catalysts were synthesized in a manner detailed by previous research [31]. These nanosheets reduce iron precipitates and activate H_2_O_2_ over a wide pH range. As well, this catalyst is efficient through multiple iterations and offers quicker oxidative degradation of recalcitrant pollutants. Various metal oxides have been widely used in catalysis [32] with manganese oxides being utilized more recently due to the Fenton-like activation of H_2_O_2_, relative abundance in the environment, and stable properties [33–35]. MnO_2_ nanomaterials are readily available through commercial means and requires no further synthesis before impregnation in AC.

A total of 12 modified AC catalysts were tested in the degradation of BZA and the production of hydroxyl radicals. Other than the raw activated carbon sample, each modified AC catalyst was modified using either of the two methods described in the modification section. The urea utilized for N-doping was purchased from Fisher Scientific, and MnO_2_ nanopowder (50 nm) was purchased from US research nanomaterials, Inc. The FeOCl nanosheets were synthesized in a manner detailed by Sun et al., 2018. If ethanol or acetone were utilized as a solvent in method 2 listed above, they are annotated in the catalyst name. Finally, AC-Mn5 and ACox-Mn5 utilized 5% MnO_2_ nanopowder to AC by weight, AC-FeOCl, ACox-Eth-FeOCl, and ACox-FeOCl-Acetone utilized 10% FeOCl to AC by weight, and much larger ratios of catalyst/AC w/w are annotated in the catalyst name if above 10%.

#### Batch experiments and analytical techniques

10 mM of BZA was utilized simultaneously for degradation experiments and hydroxyl radical production. 50 mL of 10 mM BZA, adjusted to pH 4, with 5 mM Na_2_SO_4_ electrolyte and 1 mM phosphate buffer was added to a beaker at 25°C. 10 mM H_2_O_2_, purchased from Fisher Scientific, was then added and the solution was vortexed vigorously. Finally, 100 mg of catalyst was added to the beaker. Samples were taken at 1, 5, 20, 40, and 60 minutes. These samples were filtered through 0.2 micrometer filters.

For subsequent pH tests and simulated aging tests, 20 mL of 10 mM BZA adjusted to applicable pH values by either HCl or NaOH, 5 mM Na_2_SO_4_ electrolyte, 1 mM phosphate buffer, 10 mM H_2_O_2_, and 40 mg of catalyst were added to vials and shaken vigorously for 5 minutes. The simulated aging of the catalyst consisted of placing 500 mg of catalyst in a solution of 1 M H_2_O_2_ in a plastic container, covering the container in aluminum foil to minimize light irradiation, and rotating for 7, 14, and 21 days. The container was then centrifuged at 4000 rpm for 10 minutes, drained, and then dried before testing in batch vials.

The *pzc* value determination of the AC-Mn5 catalyst consisted of the salt addition method detailed in previous work [36,37]. 0.2 grams of the AC-Mn5 catalyst was added to 40 mL of 0.1 M NaNO_3_ in nine 50-mL beakers. The pH was then adjusted to 2, 3, 4, 5, 6, 7, 8, 9, and 10 with HNO_3_ and NaOH. These samples were rotated for 24 hours to reach equilibrium. The resulting pH was then measured and the initial pH (pH_o_) vs. difference between final pH and initial pH (ΔpH) was plotted. The pH_*pzc*_ value was then determined to be where ΔpH = 0.

The benzoic acid samples were analyzed by high performance liquid chromatography (HPLC). The eluent consisted of a 1 mL/min flowrate, 20 microliter injection, and a 20% methanol/80% HPLC grade water adjusted with phosphoric acid (~pH 2.3). This eluent was sent through an ODS hypersil column with a runtime of 9 minutes. Images of the MnO_2_-coated AC were taken using scanning electron microscopy (SEM, FEI Scios Du alBeam), and elemental analysis was performed with energy dispersive spectroscopy (EDS, Oxford Instruments).

## Results and Discussion

### Heterogeneous Modified AC Catalysts Comparison

**Figure 1.**
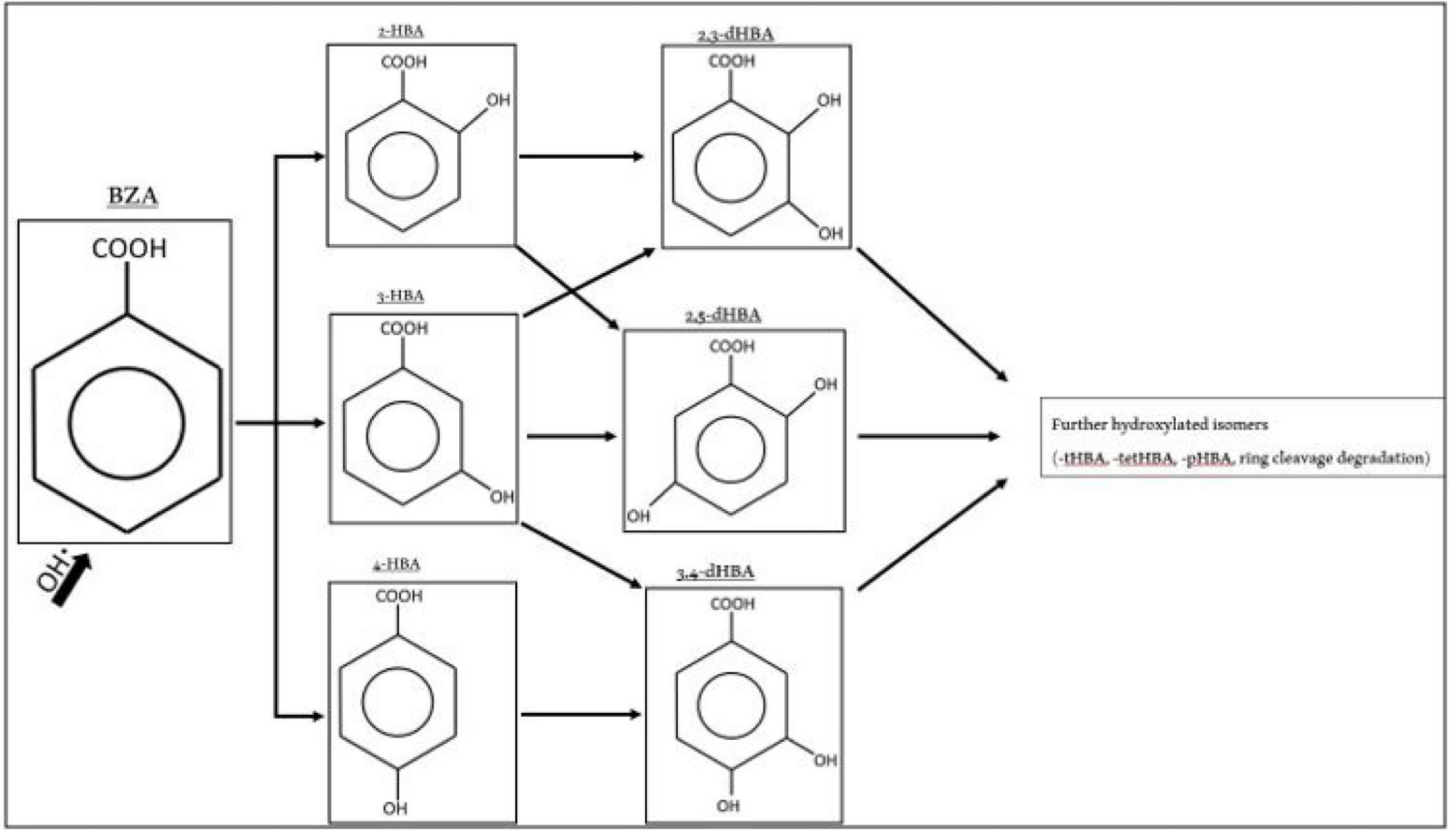
Stepwise Hydroxylation of Benzoic Acid (Oturan and Pinson, 1995)

The concentration of initial BZA and the mass of catalyst used in the experiments was to ensure that there was sufficient transformation of BZA to 4-HBA by oxidation with hydroxyl radicals to be measured in the HPLC. As hydroxyl radicals are present in the environment for a very short amount of time, it is beneficial to quantify its concentration by the byproducts of their redox reactions. BZA consists of a benzene ring with a carboxylic acid substituent and is slightly soluble in water. With the introduction of hydroxyl radicals, the unpaired electron of the oxygen can replace one of the covalently bonded hydrogen atoms at the 2, 3, or 4 positions of the benzene ring to produce *o*, *m*, or *p* – hydroxybenzoic acid, and further additional isomers (see Figure 1). The compound *p*-HBA (or 4-HBA) has a measurement sensitivity higher than other isomer byproducts, has an HPLC absorbance detection limit of ~10 nM, and is a photochemically stable byproduct of BZA oxidation with hydroxyl radicals. Most importantly, the conversion factor from 4-HBA formation to OH∙ production is 5.87 ± 0.18 [30]. This allows quantification of optimal catalytic performance from the various AC modifications by measuring the concentration of 4-HBA produced from the Fenton-like batch reactions.


Eq. 1 [OH•]=5.87[4−HBA]

The hydroxylation of BZA in the presence of an abundance of hydroxyl radicals with the primary and secondary oxidation byproducts is shown in Figure 1. For these experiments, the rate for stepwise hydroxylation is important only in the time in which samples are taken in the experimental portion. Only a few isomers of BZA oxidation byproducts are detectable at a wavelength of 254 nm wavelength in the ODS hypersil column of the HPLC. While 4-HBA is the most sensitive to detection, both 2-HBA and 3-HBA as well as 2,3-dHBA and 2,5-dHBA can be detected utilizing the analytical technique. However, 3,4-dHBA and further hydroxylated isomers – as noted in Figure 1 – are not detected with this technique. Therefore, while early quantification of 4-HBA is valid to then quantify the concentration of hydroxyl radicals produced, over the length of the experiment the rate at which 4-HBA is further oxidized and no longer detectable makes determining the precise continued catalytic activity of various modified AC materials difficult.

**Figure 2.**
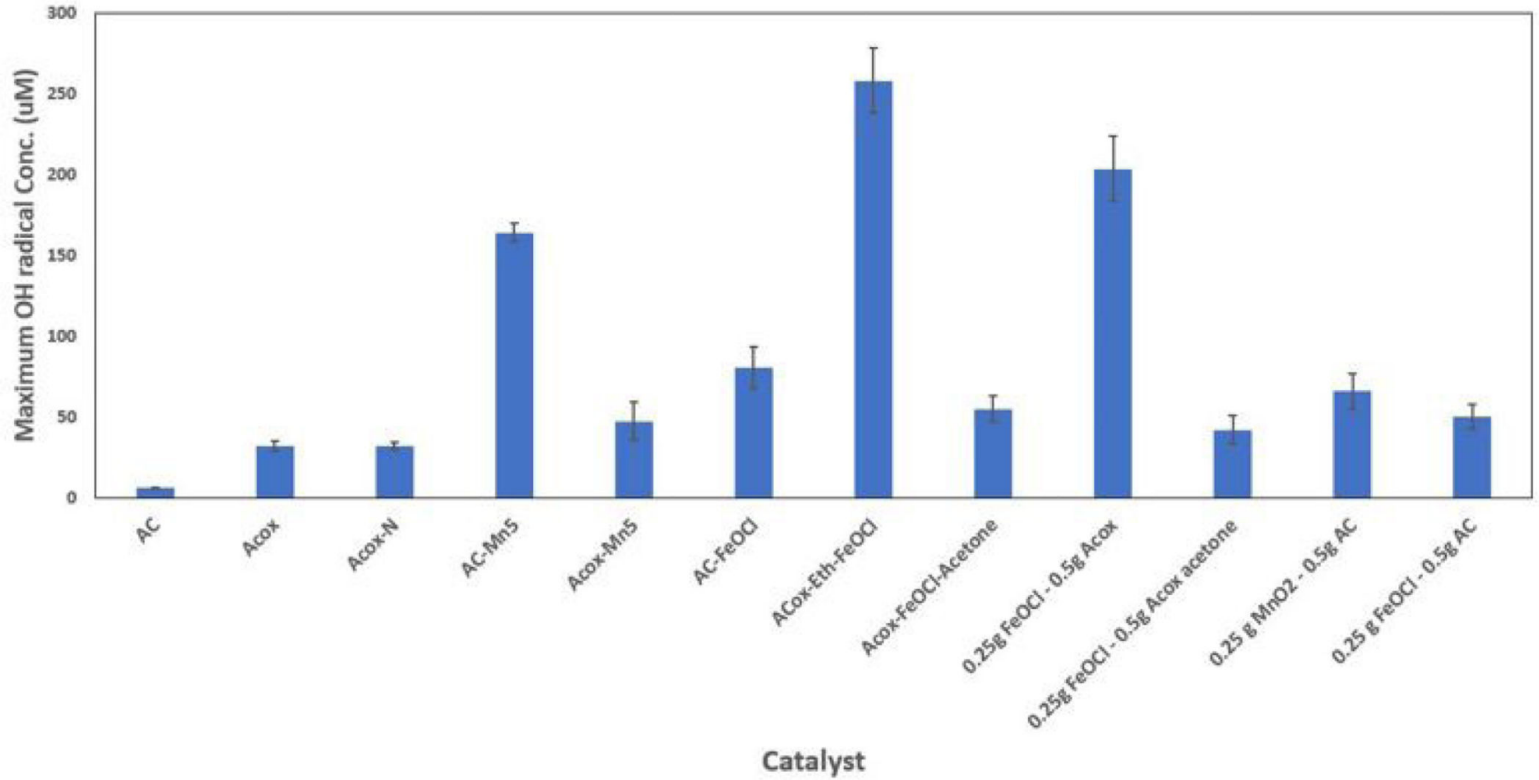
Hydroxyl Radical formation with various modified AC catalysts in the presence of 10 mM H_2_O_2_

Figure 2 shows the maximum hydroxyl radical concen tration produced from the various catalysts. Raw AC with no additives as a control did produce hydroxyl radicals, though less than all other catalysts with additives. ACox - the AC oxidized in a concentrated nitric acid solution – has an increased radical production capability due to an increased number of electron-rich base sites and radical edge sites caused by oxygen removal during this treatment [38]. The urea-doped ACox (ACox-N) performed as adequately as standard ACox. Previous research shows this ACox-N (referred to as ACN) to have very marginally better BA removal, and their main goal of N-doping was to prevent leaching of Fe ions co-doped with nitrogen groups [29]. While these nitrogen groups may indeed increase the catalytic activity of the carbon surface somewhat, in this experimental there was too little additional activation to be noted clearly. The other catalysts tested contained additives of either MnO_2_ (noted as either MnO_2_ or Mn5) or FeOCl, had a w/w ratio of 0.25g catalyst/0.5g AC(ox), and were washed in various solvents. Of these, ACox-Eth-FeOCl produced the highest concentration of hydroxyl radicals (~260 uM), followed by a w/w ratio of 0.25g FeOCl/0.5g ACox producing a hydroxyl radical concentration around 200 uM, and AC-Mn5 (AC impregnated with ~5% MnO_2_) producing nearly 170 uM. The other six catalysts produced only 75 uM OH∙ or less.

### Catalytic Mechanisms of Applied Nanomaterials

Determining certain mechanisms for hydroxyl radical formation from FeOCl catalysis as well as MnO_2_ can explain the superior catalytic performance of the previously mentioned AC modifications. The use of FeOCl nanosheets as catalysts to produce Fenton-like reactions over traditional homogenous iron catalysts is well documented. The traditional redox cycling of Fe(II)/Fe(III) in Fenton chemistry in the presence of H_2_O_2_ is the central mechanism for producing hydroxyl radicals, with the subsequent reduction of Fe(III) to Fe(II) as the limiting factor. With FeOCl nanosheets, less than 33% of iron present in the sheet takes the form of Fe(II) with Fe(III) dominating, and in the presence of H_2_O_2_ this stable Fe(III) is reduced to Fe(II) within the nanosheet [31]. Subsequent redox cycling, similar to traditional Fenton chemistry, then takes place within the FeOCl nanosheet producing hydroxyl radicals. Although shown to potentially be a powerful selective oxidant in combination with metal oxides, further oxidation to Fe(IV) is absent within this crystalline structure which has typically been associated with poor catalysis at neutral pH values [39,40]. The large surface area of the nanosheet coupled with increased electron transfer driven by the Fe-O/Fe-Cl bonds promote greater decomposition of H_2_O_2_ and formation of hydroxyl radicals [41]. Furthermore, adsorption of pollutants onto the FeOCl surface has been documented, reducing the diffusion distance of hydroxyl radicals, and increasing degradation [33]. The mechanistic catalysis of H_2_O_2_ in the presence of Fe (III/IV) in the FeOCl nanocatalyst detailed above may be described by equations 2–4 [28].


Eq. 2 $${\text{Fe}}^{3+}+{\text{H}}_{2}{\text{O}}_{2}\to {\text{Fe}}^{2+}+{\text{HO}}_{2}•+{\text{H}}^{+}$$

Eq. 3 $${\text{Fe}}^{2+}+{\text{H}}_{2}{\text{O}}_{2}\to {\text{Fe}}^{3+}+\text{OH}•+{\text{OH}}^{-}$$

Eq. 4 $${\text{H}}_{2}{\text{O}}_{2}+{\text{HO}}_{2}•\to \text{OH}•+{\text{O}}_{2}^{-}•+{\text{H}}_{2}\text{O}$$

The mechanisms involved with reactive oxygen species (ROS) formation due to decomposition of H_2_O_2_ in the presence of certain metal oxides, particularly manganese oxides, is also well documented. This heterogeneous decomposition involves multiple chain mechanisms involving free radicals, the summation of which allows for the abundant generation of radicals shown in Figure 2 [42]. There is evidence to suggest that Mn(III)/Mn(IV) ions in the presence of H_2_O_2_ produce superoxide radicals (${\text{O}}_{2}^{-}•$) which then in turn react with water and hydrogen peroxide to form hydroxyl radicals [43]. However, in systems with MnO_x_ compounds, hydroxyl radicals are significantly responsible for oxidation reactions and degradation over superoxide radicals. Indeed, the mechanisms for this process resemble that of traditional Fenton redox cycling, in that Mn(IV) is oxidized by H_2_O_2_ to Mn(III) which in turn decomposes H_2_O_2_ to a hydroxide ion and hydroxyl radical [44]. These mechanisms are made more effective with the use of crystalline modifications of MnO_2_ - nanostructures with high activity, low activation energy, and stability throughout iterative cycles [34]. These structures – nanowires, nanopowders, nanoparticles – have high surface areas and adsorptive capabilities, allowing a similar reduced diffusion distance for generated hydroxyl radicals to degrade pollutants similar to FeOCl nanosheets [45]. One additional advantage of MnO_2_ nanomaterials is the previously demonstrated scalability of synthesis while maintaining cost-effectiveness and efficiency [46]. The mechanistic catalysis of H_2_O_2_ in the presence of MnO_x_ detailed above may be described by equations 5–6 [44]. As well, equation 4 from the Fe/H_2_O_2_ reaction above is applicable with MnO_x_ catalysts as well.


Eq. 5 $${\text{Mn}}^{4+}+{\text{H}}_{2}{\text{O}}_{2}\to {\text{H}}^{+}+{\text{HO}}_{2}•+{\text{Mn}}^{3+}$$

Eq. 6 $${\text{Mn}}^{3+}+{\text{H}}_{2}{\text{O}}_{2}\to {\text{OH}}^{-}+\text{OH}•+{\text{Mn}}^{4+}$$

The AC modification with the best catalytic performance is the inertly heat-treated activated carbon doped with FeOCl nanosheets with an ethanol solvent. The method in synthesizing this catalyst further explains the increased performance over other catalysts. This ACox-Eth-FeOCl catalyst was prepared by dissolving the FeOCl nanosheets in ethanol followed by ACox.

This suspension was rapidly vortexed and subsequently rotated for 48 hours. The suspension was then poured into a quartz holder and the ethanol allowed to evaporate. These solids were then heat-treated in a tube furnace as described in the experimental. Thus, this catalyst involves doping the already impregnated FeOCl nanosheets further onto the ACox surface. In addition to this doping method and although there were no other catalysts tested which utilized ethanol as a solvent, the impact of the ACox modification on the overall catalytic performance can be suggested from comparing the performance of other AC modified catalysts. In addition to weakly acidic functional groups formed from the acidic oxidation of AC, evidence suggests the number of acidic sites on the surface of the carbon material increase while basic sites decrease when AC is either oxidized by a strong acid or heat treated under an inert atmosphere [47].

### BZA Removal/Hydroxyl Radical Production of Raw and Chemically Oxidized Modified ACs

**Figure 3.**
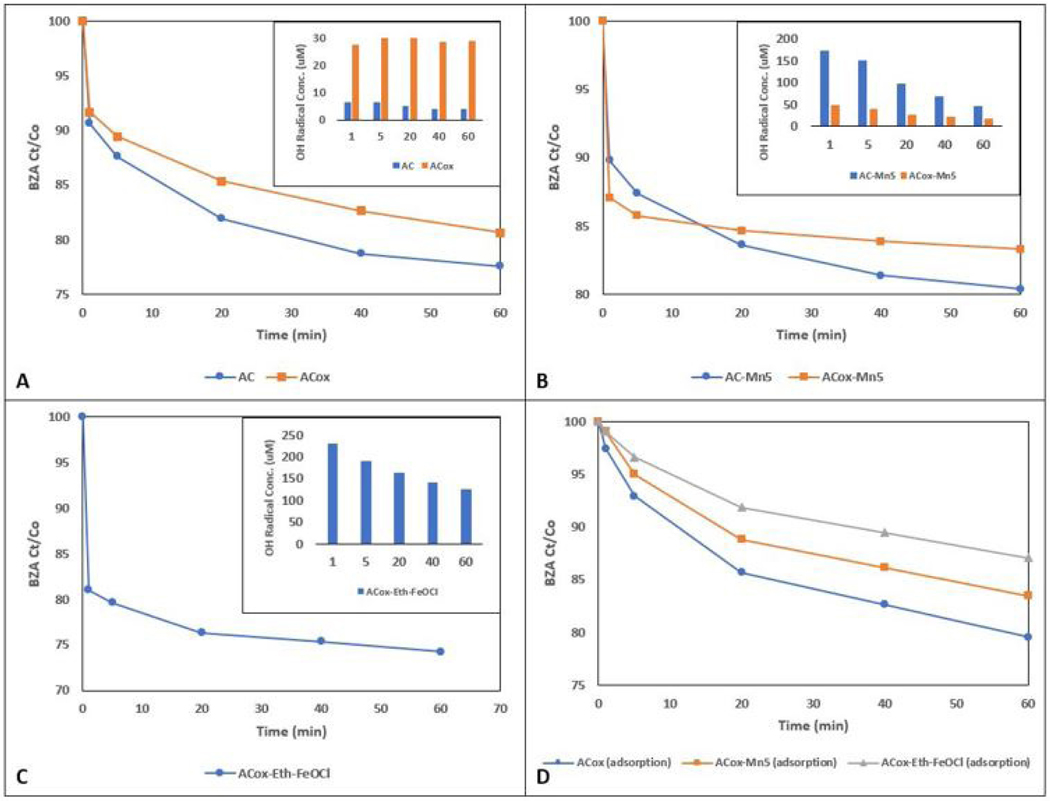
1-hr single-pot batch BZA removal and OH radical generation for (A) untreated AC and acidically-oxidized ACox, (B) MnO_2_ modified AC-Mn5 and ACox-Mn5 catalysts, (C) iron oxychloride modified ACox-Eth-FeOCl catalyst, and (D) ACox and ACox modification adsorption profiles

Figure 3 suggests this increase in acidic surface sites on oxidized AC as well, in that although ACox elicits increased hydroxyl radical generation, untreated AC elicits slightly higher BZA removal. In prior research, this ACox has been tailored for specific chemicals and particular functions due to weakly acidic functional groups formed in this process and affinity for further doping, however there is possible loss of microporosity due to clogging by humic substance by-products [48]. The difference in the hydroxyl radical formation from not only raw AC to ACox, but from 0.25g FeOCl/0.5g AC to 0.25g FeOCl/0.5g ACox in Figure 2 as well suggests the oxidized ACox produces increased catalytic activity when combined with FeOCl nanocatalysts. This may be due to the increased acidic sites and decreased basic sites resulting from this oxidation, and tracks with previously realized results of BZA degradation [29]. As well, the FeOCl nanocatalysts are highly stable and may remain highly catalytically active once impregnated or doped onto carbon surfaces. Ethanol, while a standard solvent utilized in micromaterial synthesis, here is a highly effective solvent of FeOCl nanosheets as shown in figure 2. Materials which utilize the catalytic properties of FeOCl regularly employ ethanol as a solvent of these iron oxychloride nanoparticles to disperse the compound more effectively throughout a solution to embed or impregnate onto backbones [49,50]. Although the 0.25g FeOCl/0.5g ACox sample performed exceedingly well in these single-pot hydroxyl radical formation tests, it still underperforms ACox-Eth-FeOCl while utilizing a larger mass quantity of FeOCl.

The MnO_2_ modification that produced the best catalytic performance was the AC-Mn5 sample (5% MnO_2_ nanopowder per gram AC_dw_). Noting the effect that ACox has on the FeOCl modified catalysts mentioned above and the higher generation of hydroxyl radicals from unmodified ACox, the ACox-Mn5 modification performs poorly compared to AC-Mn5. Figure 3B shows the BZA degradation and hydroxyl radical generation of both AC-Mn5 and ACox-Mn5 in the presence of 10mM H_2_O_2_. The BZA removal rate due to ACox-Mn5 is roughly 3% and 2% higher at 1 and 5 minutes of the degradation tests, respectively. However, AC-Mn5 continues to remove BZA by an additional 7% compared to 2% removed by ACox-Mn5 over the course of the test. There are multiple mechanisms at play in this removal, including hydroxylation by hydroxyl radicals, adsorption onto the carbon surface, and possible superoxide oxidation. Although there is significant removal of BZA by ACox-Mn5, there is little 4-HBA detected by analytical techniques and therefore a small amount of quantifiable hydroxyl radical formation due to ACox-Mn5 catalysis. Figure 3C shows the BZA removal due to ACox-Eth-FeOCl and shows that there is significant BZA removal within the first minute and through the course of the test (19% and 7%, respectively). However, relatively high concentrations of 4-HBA are detectable in the ACox-Eth-FeOCl test, quantifying the increased hydroxyl radical production as opposed the ACox-Mn5 test. The lack of quantifiable 4-HBA generation from ACox-Mn5 might suggest that a large portion of BZA is rapidly hydroxylated to 2,3, or 4-HBA and further hydroxylation occurs in the first minute as shown in Figure 1. However, the ability to detect high 4-HBA levels in the FeOCl modified catalysts possibly refutes this, as too does possible increased adsorption of generated 4-HBA onto the ACox surface, shown explicitly by the adsorption profiles of the various ACox modifications in figure 3D. There is a clear distinction in the ability of the various ACox modifications to adsorb BZA onto their surfaces based on the choice of catalytic material. Indeed, ACox alone adsorbs a higher mass of BZA over the course of the test than ACox modified with MnO_2_ nanomaterials, which in turn adsorbs more mass than the ethanol-FeOCl nanomaterial modified ACox (though this does involve the doping method as well). This is to be expected as impregnated and doped nanomaterials will occupy active adsorptive sites on the activated carbon surface.

Another important factor to take into consideration is the kinetic aspect of this catalytic process. Adsorption processes occurring on the catalyst surface between the contaminant species - modeled here by BZA - and the material itself are vital due to the hydroxyl radicals produced having extremely short lifespans of a few nanoseconds for oxidation; thus, degradation and removal efficiency hinges on the collision probability and chemical interaction between the contaminant and catalyst surface [51]. This kinetic heterogeneous catalytic process can be most accurately modeled by the Langmuir-Hinshelwood model, and more specifically by the apparent first-order equation 7 shown below.


Eq. 7 $$\text{ln}\left({\text{C}}_{0}/\text{C}\right)+{\text{k}}^{\prime }\left({\text{C}}_{0}-{\text{C}}_{\text{t}}\right)={\text{k}}^{\prime }\text{Kt}=\text{kt}$$

Utilizing equation 7 for the determination of rate constants of BZA removal for the catalysts shown in figure reveals additional insight into the catalytic kinetics utilizing these modified activated carbons. The overall removal rates for AC and ACox shown in figure 3A are 0.0026 min^−1^ and 0.0021 min^−1^. The rates for AC-Mn5 and ACox-Mn5 in figure 3B are 0.0018 min^−1^ and 0.0007 min^−1^, and the rate for ACox-Eth-FeOCl in figure 3C is 0.0014 min^−1^. These rates reveal several possible mechanisms occurring. The relatively higher rate constants for the activated carbons with no addition of MnO_2_ or FeOCl may be due to higher monolayer chemisorption of the BZA molecules at the solid-liquid interface, though there is much lower generation of hydroxyl radicals due to the absence of highly catalytic materials [52]. The rates for AC-Mn5 and ACox-Mn5 reveal that although there is a rapid removal of the BZA by the ACox-Mn5, there is a significantly lower overall removal rate due to the lower hydroxyl radical generation and decreased ability for chemisorption on the carbon surface compared to ACox. The ACox-Eth-FeOCl rate constant is similar to AC-Mn5, potentially due to the similar increased hydroxyl radical generation even though it has a lower BZA adsorption capability than either ACox or ACox-Mn5.

The most likely explanation for the ACox-Mn5 catalyst’s reduced and AC-Mn5 catalyst’s increased hydroxyl radical formation is due to the combination of the unique surface properties of the oxidized ACox and the MnO_2_ nanoparticles. Where the FeOCl nanosheets may indeed have more of an adsorptive capability than that of MnO_2_, leading to shorter radical diffusion distances and formation of 4-HBA, the MnO_2_ impregnated onto the adsorption-enhanced ACox possibly leads to less pore volume, adsorptive capability, and increased diffusion distance for hydroxyl radicals. This acts as a detriment to the useful formation of hydroxyl radicals that can reach BZA and form 4-HBA due to the decreased ability of H_2_O_2_ to interact with manganese ions adsorbed on the ACox structure that are no longer catalytically active. Although electron-rich base sites, edge sites, and fewer basic sites on ACox may promote higher hydroxyl radical formation by itself, in combination with MnO_2_ nanomaterials unmodified AC promotes increased oxidation of BZA by way of hydroxyl radicals and thus a more effective AC catalyst modification.

Both ACox-Eth-FeOCl and AC-Mn5 are the catalysts with the best catalytic performance that utilize either FeOCl or MnO_2_ nanomaterials, respectively. While the batch experimental technique is adequate to show a comparison of catalytic performance for a large number of modified AC catalysts, there are limitations with this method. Rapid and variable initial hydroxylation of BZA can create errors in adequately measuring 4-HBA. As well, determining the continued hydroxyl radical formation is limited due to the scavenger-like hydroxylation of the 2^nd^, 3^rd^, and 4^th^ order byproducts of BZA hydroxylation. While this scavenging resembles the actual degradation of recalcitrant pollutants and a significant obstacle in AOP implementation, it limits the ability to quantify the full effectiveness of these catalysts. This is shown in Figure 3 with the comparison of hydroxyl radical formation over the length of the test for these two catalysts.

However, ACox-Eth-FeOCl and AC-Mn5 are the most effective AC modifications tested that utilize either MnO_2_ or FeOCl. ACox-Eth-FeOCl has the highest continued catalytic performance as well as contaminant removal, as evidenced in Figure 3C. The efficacy of the AC-Mn5 catalyst is promising due to its continued performance shown in Figure 3B, as well as its ease of synthesis compared to ACox-Eth-FeOCl. As explained above, the decrease in hydroxyl radical formation is most likely not due to the declining catalytic performance of the catalysts, but rather the scavenging of hydroxyl radicals by BZA byproducts, further hydroxylation of 4-HBA that is not detected in analysis, and adsorption of 4-HBA onto the carbon surface. In addition, there is obvious rapid hydroxylation of BZA with the use of these two catalysts in the presence of 10 mM H_2_O_2_, about a third more effective for ACox-Eth-FeOCl than AC-Mn5 within the first 5 minutes of contact with the BZA/H_2_O_2_ mixture. Therefore, it is proposed that AC-Mn5 is ~66% as effective of a catalyst for hydroxyl radical formation as ACox-Eth-FeOCl. The decision point of the most optimal catalyst rests, therefore, on the goal of utilizing heterogeneous catalysts with materials and supports that are economical, relatively simple to synthesize, and scalable. While FeOCl catalysts are not toxic and relatively environmentally friendly, the complex synthesis and reduced scalability of the ACox-Eth-FeOCl catalyst severely limits this as an actionable catalytic support. As mentioned previously, the ACox-Eth-FeOCl requires chemical oxidation of the washed AC by a concentrated nitric acid solution, synthesis of the actual FeOCl nanocatalysts, impregnating these synthesized nanocatalysts on the carbon surface, and further heat treatment in a tube furnace for final FeOCl doping. The AC-Mn5 catalysts involves impregnating washed AC with commercially available MnO_2_ nanomaterials dispersed in DI water. While the higher catalytic performance of the ACox-Eth-FeOCl is desirable, AC-Mn5 is the optimal catalyst not only for its catalytic performance but it’s simple synthesis and scalability.

### H_2_O_2_ Concentration on Hydroxyl Radical Generation/BZA Removal for AC-Mn5 Catalyst

**Figure 4:**
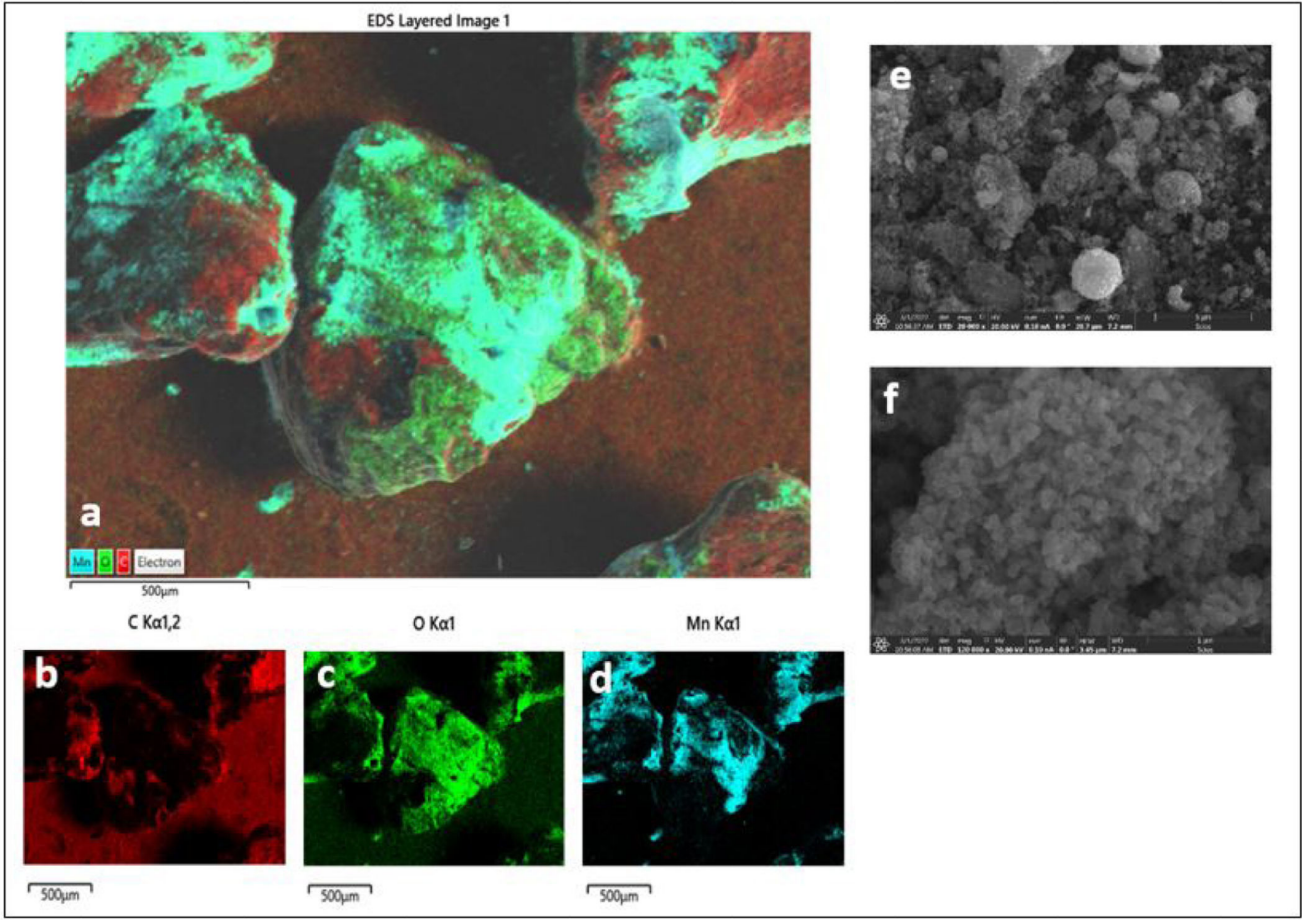
SEM images and SEM-EDS maps of GAC-Mn5 particles. Panel a is an overlay of an SEM image and the individual carbon (b), manganese (c), and oxygen (d) elemental maps. The overlapping Mn and O regions indicate MnO2. Panels e and f are higher magnification SEM images showing MnO2 aggregates and primary particles. Scale bars on panels e and f are 1 micron

**Figure 5.**
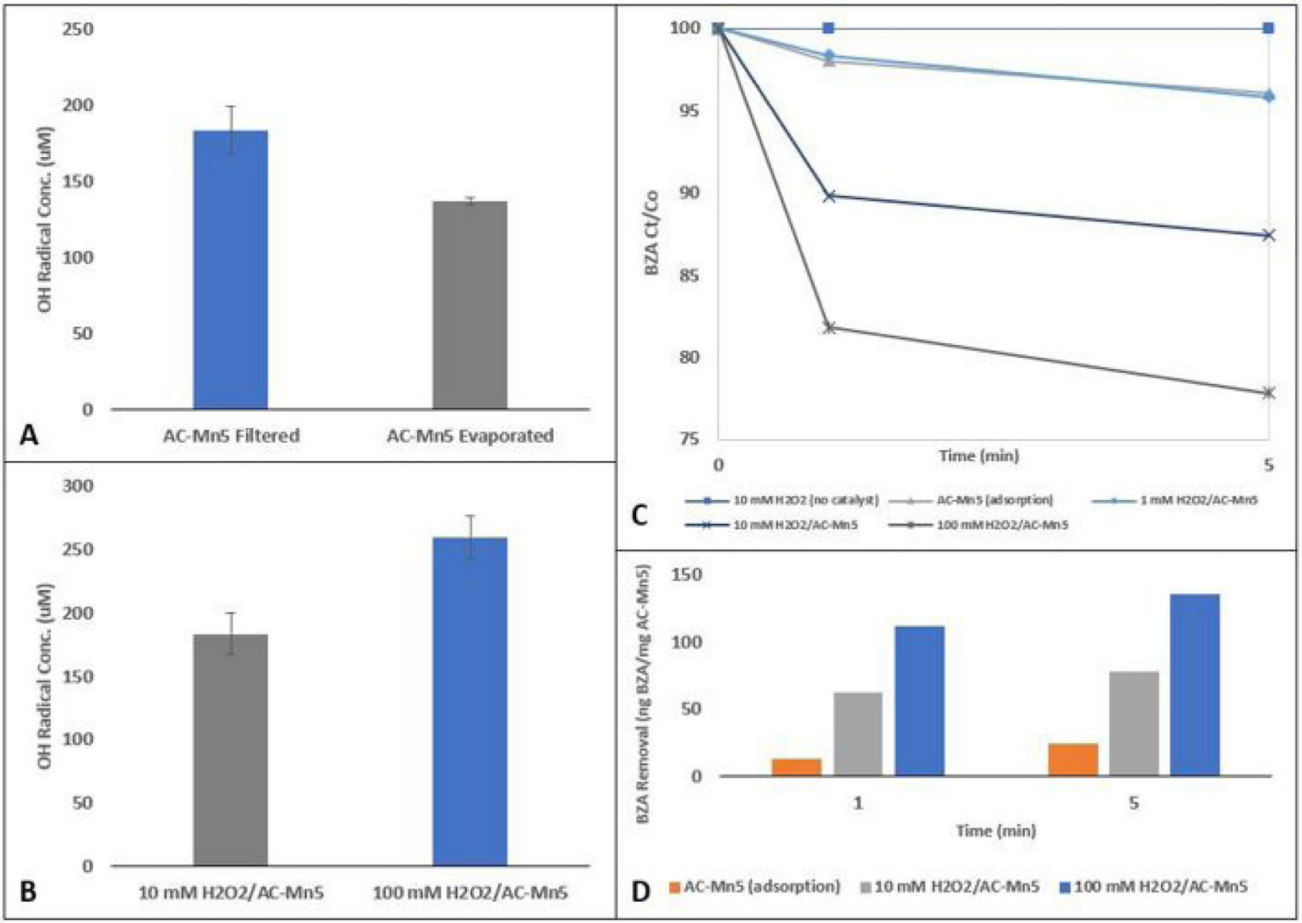
(A) AC-Mn5 synthesis variations on hydroxyl radical formation, (B) H_2_O_2_ concentration comparison on hydroxyl radical formation in the presence of AC-Mn5, (C) comparison of BZA removal with varying H_2_O_2_ concentrations, AC-Mn5 adsorption, and 10 mM H_2_O_2_ without AC-Mn5 catalyst, and (D) comparison of adsorption and increasing H_2_O_2_ concentrations on mass of BZA removed per mass AC-Mn5 catalyst

Determining the effect of AC-Mn5 preparation techniques, H_2_O_2_ concentration, pH and simulated aging further reveals the applicability of AC-Mn5 as a viable catalyst for implementation in AOP technologies. Figure 4 shows SEM-EDS images of the AC-Mn5 catalyst and reveals the presence of MnO_2_ mostly coating the AC particles. It is possible not all AC surfaces were exposed to suspended MnO_2_, or the supplied MnO_2_ was insufficient for fully coating the AC surfaces. The primary particles of MnO_2_ were confirmed to be near 50 nm in diameter at higher magnifications, and larger aggregates of MnO_2_ were commonly observed. The amount of unsupported catalytic nanomaterials (both MnO_2_ and FeOCl) may affect the catalytic performance of the various AC modifications. In figure 2, apart from 0.25g FeOCl/0.5g ACox, these increased w/w ratio modifications had poor catalytic performance compared to lower w/w ratio AC samples. One particular note on these higher w/w ratio catalysts was the amount of ‘free’ nanomaterials disassociated with the AC or ACox backbone. This characteristic of the various nanomaterials emerged from evaporating the carbon/nanomaterial slurry after vigorous mixing as opposed to filtering through a filter paper. Activated carbon as a support for MnO_2_ nanomaterials has been previously utilized, with degradation tested against unsupported MnO_2_ [53]. Enhanced degradation occurs with MnO_2_ supported on activated carbon – as seen in figure 4 - compared to unsupported MnO_2_ nanomaterials due to increased hydroxylic groups inherent to the carbon surface. As it were, the presence of unsupported nanomaterials may have an antagonistic quality in a system utilizing AC supports by scavenging H_2_O_2_ before it can reach these carbon sites with increased numbers of hydroxylic groups and not allowing it to co-locate on the carbon surface with benzoic acid to facilitate hydroxylation of the benzene ring. Figure 5A demonstrates the efficacy of the filtering technique over the evaporation technique for developing the final AC-Mn5 product.

The benefits of the filtering technique also extend to the reduced time and effort associated with synthesizing the AC-Mn5 catalyst, further actualizing the goal of this study. With the single-batch tests conducted previously, the concentration of H_2_O_2_ was constrained to 10 mM. Figure 5C shows various batch tests conducted with different initial concentrations of H_2_O_2_, as well as two controls of catalyst with no H_2_O_2_ present (adsorption tests denoted as AC-Mn5 (adsorption) in figure 5C) and 10 mM H_2_O_2_ with no catalyst present (10 mM H_2_O_2_ in figure 5C). While previous BZA removal profiles shown in figure 3 do not correlate BZA removal with hydroxyl radical formation, this is most likely due to differences in the various modified AC surface chemistries. Utilizing AC-Mn5 as the only catalyst allows for a more normalized example of BZA removal as a model to pollutant removal.

Figure 5B shows the maximum hydroxyl radical concentration formed from both the 10 mM and 100 mM H_2_O_2_ tests. Evidenced in figure 5C, H_2_O_2_ alone has undetectable levels of BZA removal at a concentration of 10 mM and the AC-Mn5 catalyst in the presence of 1 mM H_2_O_2_ is within a margin of error of adsorption by AC-Mn5 alone and therefore has no observable catalytic performance at this concentration. Conversely, 10 mM H_2_O_2_ combined with the AC-Mn5 catalyst has sufficient catalytic performance (10% BZA removal in 1 minute) and in the presence of 100 mM has exceedingly effective catalytic performance (18% BZA removal in 1 minute). This performance with increased H_2_O_2_ concentration is further portrayed by the pronounced hydroxyl radical formation of 100 mM H_2_O_2_ compared to 10 mM H_2_O_2_ in figure 5B (~45% increase). Indeed, this hydroxyl radical concentration is comparable to the formation of radicals catalyzed by ACox-Eth-FeOCl. While it is safe to assume that in the presence of 100 mM H_2_O_2_, the ACox-Eth-FeOCl catalyst would perform even more effectively than AC-Mn5, the economic/efficiency cost of utilizing this high concentration of H_2_O_2_ in addition to the complex synthesis of ACox-Eth-FeOCl is unsuitable for scalability and applicability.

Figure 5D presents a mass per mass removal rate of BZA to the AC-Mn5 catalyst due to simple adsorption as well as in the presence of increasing H_2_O_2_ concentrations. The mass of BZA removed by the 1mM H_2_O_2_ and AC-Mn5 reaction is not included as it is in the margin of error of adsorption alone by the AC-Mn5 catalyst. While the numbers themselves are based on the mass of catalyst in the volume of BZA, comparing the different normalized mass removal profiles elicit more information about the role and rate of H_2_O_2_ concentrations in catalysis. Over simple adsorption, the 10mM H_2_O_2_/AC-Mn5 mixture is over 500% as effective within 1 minute of catalysis while the 100mM H_2_O_2_/AC-Mn5 mixture is over 900% as effective. Though the 100mM H_2_O_2_/AC-Mn5 mixture is over ninefold greater at removing BZA than adsorption, it is only ~75% more effective than the 10mM H_2_O_2_/AC-Mn5 mixture while utilizing 10x the concentration of H_2_O_2_. Therefore, the tenfold increase in H_2_O_2_ does not cause a linear increase in hydroxyl radical production or BZA removal in this case after the first minute during which the majority of catalysis occurs. Within the 5-minute timeframe, the 10mM H_2_O_2_/AC-Mn5 mixture is slightly over 300% as effective and the 100mM H_2_O_2_/AC-Mn5 mixture is around 550% as effective at BZA removal as adsorption alone.

There are several mechanisms occurring within this system with the addition of only H_2_O_2_ and with increased concentrations of H_2_O_2_. Since benzoic acid exists at ambient conditions with a pH<5, there is limited formation of additional H_2_O_2_ and HO_2_ radicals due to the reaction of generated hydroxyl radicals with OH^−^, leading to enhanced opportunity for benzoic acid hydroxylation by hydroxyl radicals [54]. Hydroxyl radicals have about 1.58x more oxidizing strength than H_2_O_2_ alone, leading to the conclusion that the addition of H_2_O_2_ alone is insufficient to reduce the concentration of benzoic acid [55]. However, this research shows that at higher concentrations of hydrogen peroxide in the presence of a catalyst, H_2_O_2_ adsorbed on the catalyst surface may scavenge hydroxyl radicals to produce HO_2_ radicals which are less powerful oxidants [56]. Ultimately, increasing the concentration of H_2_O_2_ in the presence of the AC-Mn5 catalyst enhances oxidation to a point and then becomes inhibitory due to the above stated reaction, leading to a conclusion that there is an optimal H_2_O_2_ concentration, as well as a sufficient H_2_O_2_ concentration to produce desirable hydroxyl radical concentrations while maintaining cost-effectiveness and the possibility for scalability [23].

These results allow a glimpse into the applicability of this type of catalysis for use in an engineered system. While 100 mM external H_2_O_2_ is a very high concentration to be scaled effectively for a large volume of pollutant treatment, 10 mM H_2_O_2_ and possibly slightly lower concentrations may be sufficient to effectively generate a large enough amount of hydroxyl radicals to oxidize recalcitrant pollutants. In addition, certain technologies and materials to *in-situ* generate H_2_O_2_ approach this 10 mM range to be even more economical and sustainable.

The increase in catalysis with a stronger H_2_O_2_ concentration can be attributed to the mechanisms detailed in equations 4–6, as there is more activity in the Mn(III)/Mn(IV) redox cycling as well as the possible reaction with hydroperoxyl radicals (weaker oxidants) to produce hydroxyl radicals. The increased activity of interaction between H_2_O_2_ and the catalyst due to higher H_2_O_2_ concentration leads to an increased hydroxyl radical formation and rapid hydroxylation of BZA in the system.

### Effects of pH and Aging on AC-Mn5’s Catalytic Capability

**Figure 6.**
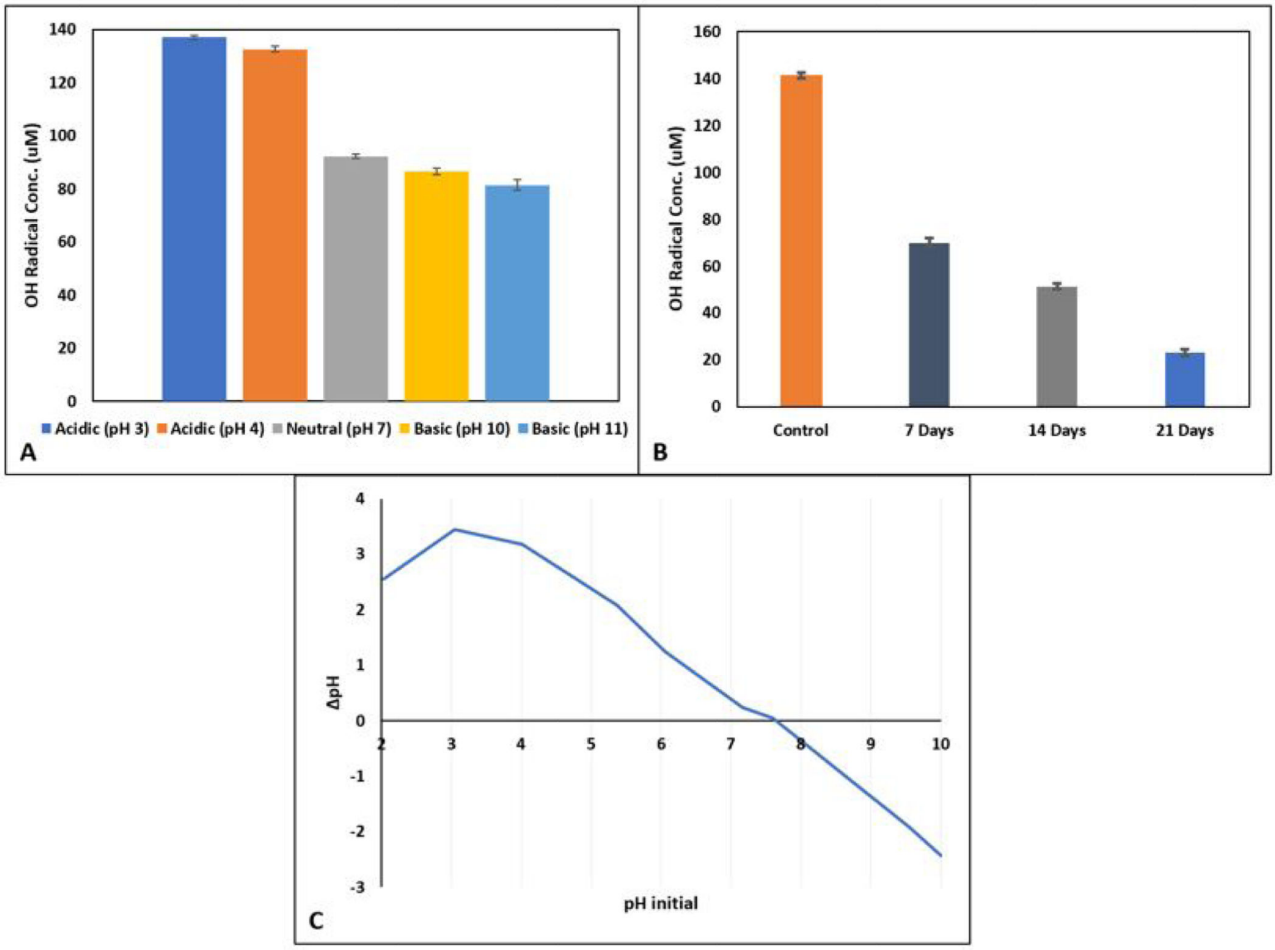
(A) Hydroxyl radical formation at varying pH values, (B) hydroxyl radical formation due to aging with 1M H_2_O_2_, and (C) pH_pzc_ (point of zero charge) determination for AC-Mn5 catalyst

The solution environment also influences the efficacy of AC-Mn5 catalytic performance, shown in figure 6. Of note, these experimental results shown in figure 6 were conducted with the evaporated AC-Mn5 catalyst as opposed to the filtration preparation utilized previously. Catalysis of H_2_O_2_ performed more effectively at acidic pH values compared to neutral and basic pH values in this test, with neutral and basic pH values comparatively similar. While the rate of the catalyst’s redox potential is inhibited in neutral and basic environments, potentially caused by the partially disassociation of H_2_O_2_ into H^+^ and HO_2_^−^ [42], it has been observed that decomposition of H_2_O_2_ overall increases with increasing pH values due to negative surface sites [57]. However, pH influences the oxidation potential of hydroxyl radicals as well as the electrostatic charges of the pollutant species. Indeed, in acidic pH environments, catalyst surfaces can become positively charged which may reduce the adsorption of certain pollutant species that are electrostatically charged due to increased repulsive forces [54]. The pK_a_ of pollutant species also play a major role in that in protonated and deprotonated forms, at pH values below and above its pK_a_ value, respectively, attractive forces between free electron pairs and catalyst surfaces can change leading to increased or decreased distances between the catalyst/contaminant and ability for enhanced oxidation by hydroxyl radicals [4].

This oxidation efficiency, the deleterious effects of the pH environment on BZA, the increased rate of the reactions, attractive/repulsive forces, as well as the disassociation of H_2_O_2_ may explain why the formation of hydroxyl radicals is decreased at more basic pH values. As explained in the experimental, samples were taken at the 1- and 5-minute marks, as these have shown to exhibit the highest concentration as 4-HBA and therefore of hydroxy radicals. Therefore, further experimentation to determine the temporal distribution with which catalytic performance is dependent on solution pH will determine the time-frame efficacy of utilizing this method for degradation experiments. In fact, various engineering solutions could be implemented that take these reaction rates into consideration to implement the most effective design for degradation of pollutants of concern. This is beneficial in considering the wide pH range applicability of this catalyst compared to the acidic pH-restrained environments normally associated with iron catalysts.

Recent research has elicited the benefit of determining the point of zero charge, pH_*pzc*_, of various catalysts. The catalyst surface charge in conjunction with the protonated or deprotonated form of the pollutant species affects attractive/repulsive forces that allow for closer contact of the contaminant with generated hydroxyl radicals for the potential for oxidation. A catalyst surface has a particular pH, or concentration of protons in solution, at which it will have a surface charge of zero, deemed the pH_*pzc*_. At solution pH values below this point, the surface will adsorb additional protons and accumulate a net positive charge, while in a solution with pH above this point the surface will adsorb hydroxyl anions and accumulate a net negative charge [58]. As such, contaminant species may exist in solution in protonated or deprotonated form depending on their disassociation above and below pK_a_ values. Where protonated there will be a repulsive force between the contaminant species and the catalyst surface at a solution pH below the pH_*pzc*_, and where deprotonated there will be a repulsive force between the contaminant species and the surface of the catalyst above the pH_*pzc*_ [59]. As benzoic acid has a pK_a_ value of 4.2, around 80% is in its anionic form of benzoate at its natural pH of 3.7. Based on figure 6C, the pH_*pzc*_ of the AC-Mn5 catalyst is estimated to be 7.6, with a positive charge below this value and negative charge above this value. Therefore, the interaction between the AC-Mn5 catalyst below this pH_*pzc*_ and below the pK_a_ of the contaminant species lead to greater electrostatic adsorption interactions. This decreases the distance between the catalyst surface the and benzoic acid, leader to a greater capacity for hydroxylation by hydroxyl radicals. Above the pH_*pzc*_ and where benzoic acid is fully in its anionic form, there is electrostatic repulsion between the contaminant species and catalyst surface and a decrease in the hydroxylation by hydroxyl radicals. This is suggested by the higher hydroxyl radical ‘generation’ in figure 6A at lower pH values and supported by findings in previous research for benzoic acid and carbonaceous materials [60]. Ultimately, this suggests that while the hydroxylation of benzoic acid decreased with increasing pH, since hydroxylation has been utilized in this research as an indicator of total hydroxyl radical concentration the AC-Mn5 catalyst may be even more effective over a wider pH range with other contaminants that dissociate at higher pH values.

To determine the effect of the AC-Mn5 catalyst’s longevity as well as higher H_2_O_2_ concentration, experiments were conducted as indicated in the experimental and results shown in figure 6B. The catalyst, when ‘aged’ with a 1M solution of H_2_O_2_ has ~50% reduced catalytic performance compared to the control after 7 days of rotation. After an additional 7 days of contact with the 1M H_2_O_2_ solution, the catalyst has ~75% reduced catalytic performance than the control, and nearly 90% reduced performance after 21 days. These samples were covered to reduce the possibility of photolytic degradation of the H_2_O_2_. The reasons for this reduced performance after the 7-, 14-, and 21-day periods could be the reduction of catalytic edge sites, disassociation of MnO_2_ from the AC matrix, decomposition of MnO_2_ nanosheets, and reduction of adsorptive capabilities of the nanosheets themselves. Of note, this treatment with strong H_2_O_2_ concentrations has been utilized to increase AC adsorption and resembles the ACox treatment with concentrated HNO_3_ [61]. This may further explain not only the reduced capability of the AC-Mn5 after simulated ‘aging’ but show how this type of treatment may cause the reduced catalytic performance seen with the ACox-Mn5 modification. The minimization of the ability for H_2_O_2_ to co-locate with MnO_2_ nanoparticles at the edge sites may lead to this reduced catalytic performance, in addition to disassociation of the nanomaterials from the carbon surface itself and resembling similar effects seen in figure 5A [62–66]. The reduction in the adsorptive capabilities due to possible nanomaterial decomposition or modifying the surface charge of the catalyst removes the synergistic effects of decreased diffusion distance for hydroxyl radicals to hydroxylate BZA, possibly causing this reduction. However, this ‘aging’ simulates the applicability of this catalyst over numerous iterative cycles, and although reduced, the AC-Mn5 modification maintains adequate catalytic performance. Indeed, further applicable longevity tests in optimized mediums may further indicate the efficacy of this catalyst modification for real-world pollutant degradation.

## Conclusion

A research pathway was proposed to choose a heterogeneous catalyst which elicited Fenton-like reactions in the presence of H_2_O_2_ with a focus on utilizing materials and supports that are economical, relatively simple to synthesize, and scalable. A number of samples were synthesized with activated carbon as the central backbone of the supporting material. Two methods for either doping or impregnating the carbon backbone were utilized, as well as two main nanocatalysts in the form of FeOCl and MnO_2_. Batch experiments were conducted with 10 mM BZA, 10 mM H_2_O_2_, and 100 mg of catalyst at pH 4 and 25°C to determine both the removal of BZA and hydroxylation of benzoic acid to 4-HBA by produced hydroxyl radicals. Of the FeOCl catalysts tested, the catalyst ACox-Eth-FeOCl, heat-treated ACox doped with ethanol-wash FeOCl nanosheets, had the best catalytic performance in producing hydroxyl radicals. The catalyst AC-Mn5, unmodified AC impregnated with 5% MnO_2_, had the best catalytic performance of the MnO_2_ modified catalysts tested. The ACox-Eth-FeOCl is roughly 33% more effective at producing hydroxyl radicals than AC-Mn5, and both had similar hydroxyl radical production profiles over the course of the tests conducted. AC-Mn5 is the best candidate based on the proposed research pathway due to its sustainability, economy of scale, and ease of synthesis compared to the labor-intensive catalyst ACox-Eth-FeOCl. Multiple subsequent tests were conducted to demonstrate the effect of AC-Mn5 synthesis modifications, performance in various concentrations of H_2_O_2_, catalytic effectiveness in varying pH regimes, and longevity of the catalyst by simulated aging. AC-Mn5 was more effective when filtered as opposed to evaporated after slurry mixing, produced both higher production of hydroxyl radicals and BZA removal with increased H_2_O_2_ concentrations, has increased reaction rates in acidic pH regimes, and has adequate potential for hydroxyl radical production with iterative cycles. Future research should focus on engineering designs that best implement this catalyst into a resilient, robust, and maintainable system for the degradation and removal of recalcitrant pollutants by heterogeneous Fenton-like reactions.
